# Successful Treatment of Localized Pemphigus Foliaceus with Topical Pimecrolimus

**DOI:** 10.1155/2013/489618

**Published:** 2013-09-17

**Authors:** G. Tyros, K. Kalapothakou, E. Christofidou, A. Kanelleas, P. G. Stavropoulos

**Affiliations:** ^1^1st Department of Dermatology and Venereology, “A. Sygros” Hospital for Skin and Venereal Diseases, University of Athens School of Medicine, 5 Dragoumi Street, 16121 Athens, Greece; ^2^Histopathology Department, “A. Sygros” Hospital for Skin and Venereal Diseases, 5 Dragoumi Street, 16121 Athens, Greece

## Abstract

We report the case of successful treatment of a 79-year-old male patient with recurrent pemphigus foliaceus with pimecrolimus cream 1% once daily for 40 days. The patient initially presented with localized lesions on the scalp and nose area and was treated with systemic corticosteroids. At his fourth relapse within a period of 16 months, he refused any systemic treatment. Pimecrolimus cream was suggested to him as an alternative option.

## 1. Introduction

Pemphigus foliaceus (PF) is a chronic autoimmune blistering disease mainly affecting the cornified skin of the face and upper torso, such as the presternal and interscapular regions, rather than the lower torso or the scalp. PF, as well as pemphigus vulgaris (PV), is characterized by the loss of subcorneal keratinocyte cell adhesion. The latter is clinically expressed by the formation of fragile vesicles which rupture easily, leaving behind erosions. In PF, pathogenic immunoglobulin G (IgG) targets the desmosome cadherin desmoglein1, a 160 KDa, calcium-dependent, transmembrane glycoprotein that plays an important role in cell-to-cell adhesion of the most differentiated epidermal epithelia.

 Most cases of PF are treated with systemic glucocorticosteroids with or without immunosuppressive therapy, although some mild cases can respond well to topical glucocorticosteroids alone [1]. We report a case of PF with recurrent localized lesions at the face and scalp area, which was successfully treated with topical pimecrolimus.

## 2. Case Report

**Figure 1:**
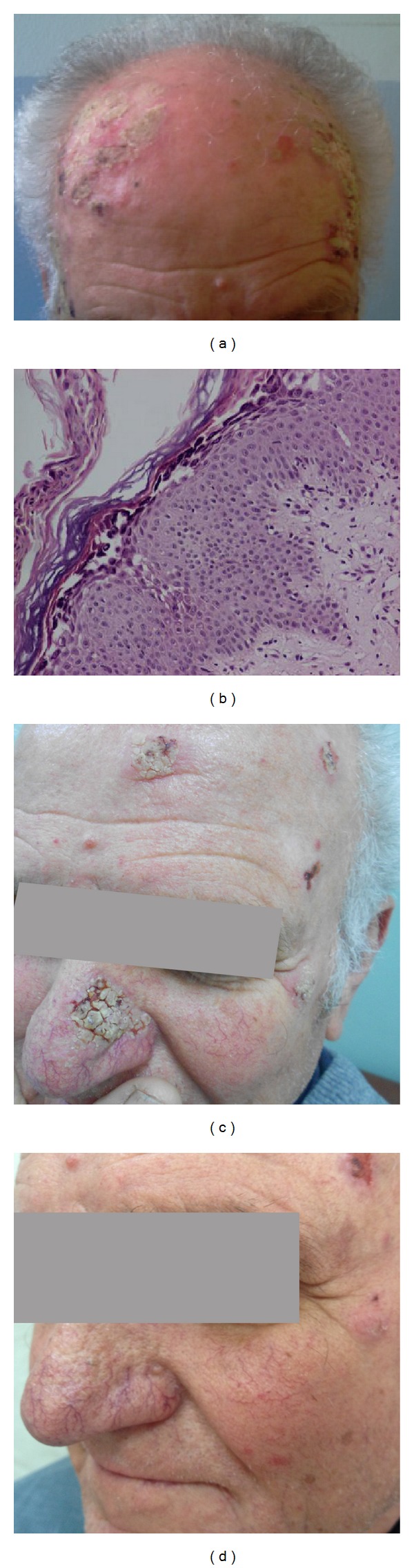
(a) Markedly hyperkeratotic scaling and erosions on scalp. (b) Subcorneal vesicle with a small number of dyskeratotic granular cells (H&E ×40). (c) Erosions and hyperkeratotic, cobblestone-like crusting on the nose of the same patient. (d) Clearance of lesions six months after the end of the pimecrolimus course.

We reviewed a 79-year-old patient in the outpatient department of our hospital. He presented with scalp erosions which had been covered with markedly hyperkeratotic scaling for the last 4 months (Figure 1(a)). He also reported mild pruritus. No previous trauma, surgery, irradiation, or any topical treatment preceded the appearance of these lesions. The lesions had been treated unsuccessfully in the past with cryotherapy. From his previous medical history, he reported rosacea of the cheeks under treatment.

A skin biopsy from the scalp was performed (Figure 1(a)). The histology report revealed features of pemphigus foliaceus, such as formation of superficial bullae with acantholytic cells, parakeratosis, acanthosis, and slight spongiosis (Figure 1(b)). The diagnosis was confirmed by direct and indirect immunofluorescence (ELISA and immunoblot), while tests for antinuclear antibodies (ANA) were negative. 

He was treated with topical betamethasone 0.05% twice daily and prednisolone 0.7 mg/kg daily for 20 days tapered to 5 mg daily over the course of the following four weeks. There was significant improvement of the lesions with complete clearance maintained for more than ten months. Subsequently, there were two relapses treated similarly as above, and our patient relapsed again sixteen months after the original diagnosis, with development of novel hyperkeratotic lesions on the nose and the scalp. The clinical features of the lesions (Figure 1(c)) were similar to the one biopsied, and therefore no other histopathologic exam was considered. This time he refused any systemic treatment, and we advised alternatively pimecrolimus cream 1% once daily at the hyperkeratotic nose lesions. The patient had been using pimecrolimus cream intermittently for his telangiectatic rosacea of the cheeks. After 4 weeks, the erosions on the nose and scalp healed, leaving postinflammatory hyperpigmentation that vanished completely after a few months (Figure 1(d)). A burning sensation during the first days of use was reported, but the medication was well tolerated after that.

## 3. Discussion

Pemphigus is a group of chronic, tissue-specific, antibody-mediated, and autoimmune blistering diseases. It is divided into three major forms: pemphigus vulgaris, pemphigus foliaceus, and paraneoplastic pemphigus. Usually it manifests as a generalized disease, whereas initial localized presentation of pemphigus is less common [2]. Before the advent of systemic corticosteroids, pemphigus was fatal in 60% of patients. Early detection of the disease has proved beneficial as late diagnosis may be implicated with severe complications (as in the erythrodermic form). So far only a few cases of limited scalp localization of PF exist in the literature [3]. Systemic corticosteroids are the mainstay of treatment for pemphigus. They are often combined with immunosuppressive agents, in order to reduce their significant side effects. Topical treatment can be used supplementary to systemic treatment or as a sole treatment in localized disease. 

Damage mechanisms by which pemphigus autoantibodies lead to acantholysis and apoptosis of keratinocytes are not yet precisely known. However, current investigation highlights the presence of autoreactive T and B lymphocytes and alterations in the immune regulation against cutaneous antigens in genetically susceptible individuals [4]. In accordance with this notion, emerging immunological advances reveal that T-helper cells are essential for maximal antibody production [5]. 

Pimecrolimus is an ascomycin macrolactam derivative with immunomodulatory functions. It has been shown in vitro that pimecrolimus binds to macrophilin-12 (also referred to as FKBP-12) and prevents the activation of nuclear factor of activated T cells (NFAT) by inhibiting calcineurin [6]. The latter is an important factor in the intracellular signal transduction pathway resulting in suppression of T cells, inhibition of the production, and release of inflammatory cytokines such as IL-2, IL-3, IL-4, granulocyte-macrophage colony-stimulating factor (GM CSF), tumor necrosis factor *α* (TNF*α*), and interferon-*γ* (IFN*γ*). Pimecrolimus also prevents the release of inflammatory cytokines and mediators from mast cells but not from Langerhans cells as does Tacrolimus [6, 7]. However, some of these cytokines have also been implicated to directly increase keratinocyte fragility in the aetiology of pemphigus vulgaris lesions [8]. We speculate that the same T lymphocyte-dependent processes might be implicated in PF, and it is through the inhibition of these that pimecrolimus seems to have benefited our patient.

Topical calcineurin inhibitors have already been established in the treatment of atopic dermatitis, and they are being used in an increasingly wide range of other dermatologic conditions. There are limited reports in the literature of tacrolimus topical use in the treatment of antibody-mediated autoimmune diseases such as PV [9] or PF [10]. However, we have found no report of pimecrolimus topical use as PF treatment.

## 4. Conclusion

Pimecrolimus appeared to be a safe and successful alternative treatment to topical corticosteroids for our patient with localized PF. Topical calcineurin inhibitors have been used sporadically for this indication in the past. Further work is needed to determine the place of topical calcineurin inhibitors in the management of PF.
